# Study on the Structure and Properties of Silk Fibers Obtained from Factory All-Age Artificial Diets

**DOI:** 10.3390/ijms25116129

**Published:** 2024-06-01

**Authors:** Mengyao Pan, Kexin Jiang, Yuwei Jin, Ying Mao, Wangyang Lu, Wenbin Jiang, Wenxing Chen

**Affiliations:** 1 College of Textile Science and Engineering (International Institute of Silk), Zhejiang Sci-Tech University, Hangzhou 310018, China; myfighting99@163.com (M.P.); 202120201018@mails.zstu.edu.cn (K.J.); jyw1026438834@163.com (Y.J.); luwy@zstu.edu.cn (W.L.); 2 National Engineering Lab for Textile Fiber Materials & Processing Technology, Zhejiang Sci-Tech University, Hangzhou 310018, China; wxchen@zstu.edu.cn; 3 Zhejiang Provincial Innovation Center of Advanced Textile Technology, Shaoxing 312000, China

**Keywords:** factory all-age artificial diets, silk fibroin fibers, structural characteristics, mechanical properties, in vitro degradation

## Abstract

The traditional production mode of the sericulture industry is no longer suitable for the development requirements of modern agriculture; to facilitate the sustainable development of the sericulture industry, factory all-age artificial diet feeding came into being. Understanding the structural characteristics and properties of silk fibers obtained from factory all-age artificial diet feeding is an important prerequisite for application in the fields of textiles, clothing, biomedicine, and others. However, there have been no reports so far. In this paper, by feeding silkworms with factory all-age artificial diets (AD group) and mulberry leaves (ML group), silk fibers were obtained via two different feeding methods. The structure, mechanical properties, hygroscopic properties, and degradation properties were studied by Fourier transform infrared spectroscopy (FTIR), X-ray diffraction (XRD), and thermogravimetric analysis (TGA). Structurally, no new functional groups appeared in the AD group. Compared with the ML group, the structure of the two groups was similar, and there was no significant difference in mechanical properties and moisture absorption. The structure of degummed silk fibers is dominated by crystalline regions, but α-chymotrypsin hydrolyzes the amorphous regions of silk proteins, so that after 28 d of degradation, the weight loss of both is very small. This provides further justification for the feasibility of factory all-age artificial diets for silkworms.

## 1. Introduction

Silk is an important biological protein polymer in which liquid silk proteins undergo a conformational transition from the spinning tubes of the silkworm to form solid fibers [1]. Natural silk fibers consist of two main protein components, silk fibroin (72–81%) and silk sericin (19–28%), forming a core–shell structure [2]. Silk fibroin acts as the core structure of a semicrystalline material to provide mechanical properties, while silk sericin acts as a globular protein that binds the two fibroin fibers together [3]. In recent years, due to the excellent mechanical properties [4], stable biocompatibility [5], tunable biodegradability [6], low immunogenicity, and low toxicity of silk proteins [7], the application of silk has no longer been limited to the textile industry and has been further extended to biomedical fields. Since sericin could cause inflammatory reactions in the body, silk fibroin and silk sericin are usually separated [8]. Degummed silk fibers have received increasing attention as a biomaterial, especially in wound-healing materials [9,10] and bone tissue engineering [11,12,13,14].

However, the sericulture industry has been limited in its continued growth and development. This is mainly because the Bombyx mori silkworm, as an oligophagous insect, feeds mainly on mulberry leaves [15]. Mulberry leaf production is limited by seasonality [16], and the high labor intensity and extremely low degree of mechanization and automation have led to a serious threat to the development of the traditional sericulture industry. To solve these problems, many researchers in Japan and China have been working on the research and application of artificial feed-based silkworm-rearing technology. In the 1970s, Japan’s feeder silkworms reached practicality and small silkworm feeder technology began to be promoted, but large-scale production was not achieved. In the 1980s, Chinese researchers also conducted a lot of studies on the effects of artificial diet on the growth of silkworms [17]. Finally, in 2019, Zhejiang Babei Group Co., Ltd. realized the industrialized development of all-age artificial diet sericulture, which is the world’s first case of industrial sericulture with all-age artificial diets, subverting the traditional sericulture model that has not changed for 5000 years. The feed used in the factory for sericulture is mainly soybean meal, corn flour, mulberry leaf powder, and vitamin complex. Its emergence not only provides a large-scale raw material cocoon guarantee for the silk industry but also realizes a high-efficiency modern cocoon silk industry production model, which has deep scientific research and expansion value.

Artificial diets for silkworms have been studied by a wide range of scholars. Zhou et al. [18] found that artificial diet affects the immune system, the digestion and absorption of nutrients, energy metabolism, and other types of protein expression related to the silk synthesis of silkworms. Chen et al. [19] have shown that glycine improves the efficiency of amino acid conversion to silk proteins. Dong et al. [20] compared the metabolomic differences in hemolymphs between full-grown artificial feed-fed and mulberry leaf-fed silkworms and found differences in the metabolism of glycine, carbohydrates, etc., which may be related to the efficiency of silk protein synthesis. Most of the current studies on the differences between artificial diet feeding and mulberry leaf feeding in Bombyx mori have focused on growth and development [21,22,23] and nutrient uptake metabolism [24,25,26]. The exploration of the secondary structure and properties of silk from silkworms fed factory all-age artificial diets has not been reported.

In this paper, we investigated the effects of two different feeding methods on the structure and performance of silk fibers, which is of great practical significance for accelerating the popularization and application of the new technology of factory all-age artificial diets for Bombyx mori silkworms.

## 2. Results and Discussion

### 2.1. Morphology of Silk Fibers

**Figure 1:**
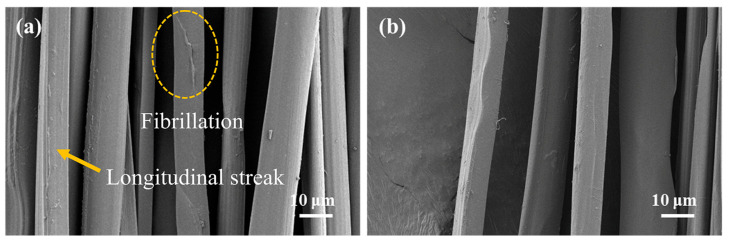
SEM images of silk fibers with two different feeding methods. (**a**) ML group, (**b**) AD group.

The surface morphology of degummed silk fibers obtained from the two feeding methods is shown in Figure 1. It is obvious that the surface of silk fibroin fibers was smooth after steam degumming treatment. Silk sericin, which was easily soluble in hot water, dissolved under the action of steam heat and humidity. After washing, the silk fibroin, which was originally adhered to by the sericin, had no adhesive force and separated. Therefore, both of them were in a dispersed state. However, the silk fibroin fibers obtained by mulberry leaf (ML group) feeding showed slight fibrillation on the fibroin fiber surface, and fine longitudinal streaks of fibrillar structures began to appear on some of the silk fibroin fibers. This may have been due to the low sericin content in the silk from the mulberry leaf feeding group, which made it faster to remove sericin in the degumming process, and then, it would have acted on the silk fibroin fiber to form split fibers. Moreover, the scanning electron microscopy (SEM) results also showed that the fineness of the degummed silk fibers in the ML group was larger than that in the AD group. The average size of silk fibers was 3.18dtex in the ML group and 2.97dtex in the AD group. Qu et al. [27] suggested that this was caused by the difference in silk protein content per unit length.

### 2.2. Effect of Factory All-Age Artificial Diet Feeding on the Structure of Silk Fibers

**Figure 2:**
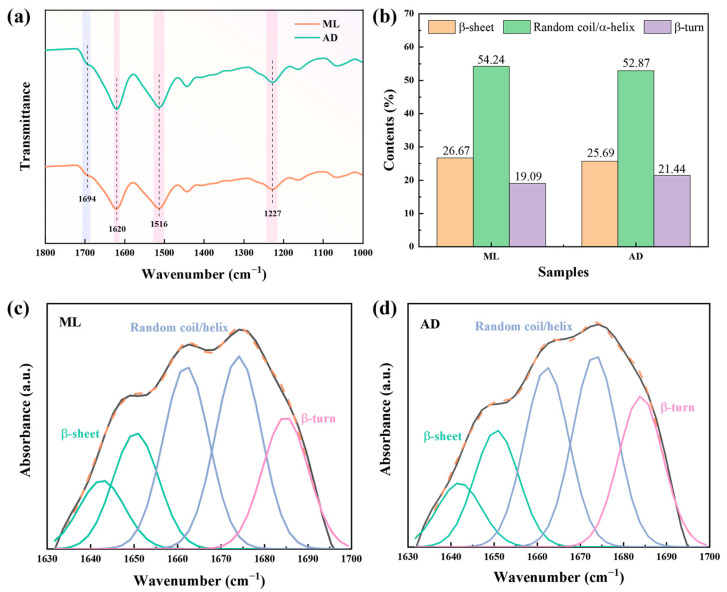
(**a**) ATR-FTIR spectra of silk fibers with two different feeding methods, (**b**) secondary structure content, (**c**) deconvolution details of amide I band of ML group, (**d**) deconvolution details of amide I band of AD group. Black line, original spectra; orange dash line, simulated spectra from the total peaks.

Analyzing the structure of the silk fibroin fibers is useful for understanding its properties. The conformational differences in protein chains and the structural changes in aggregates between mulberry leaf and artificial feed silk fibroin fibers were investigated by Fourier transform infrared spectroscopy (FTIR) and X-ray diffraction (XRD). As shown in Figure 2a, FTIR can reflect the structure of and changes in molecules, can be used to analyze the structure and conformation of silk proteins, and is often used to observe many complex amide bands of silk proteins [28], which are mainly divided into three main vibration bands, in the range of 1600 to 1700 cm^−1^ for amide I (C=O stretching), 1500 to1580 cm^−1^ for amide II (in-plane N-H bending and C-N stretching), and 1200 to1300 cm^−1^ for amide III (N-H bending and C-N stretching) [29]. The frequency splitting observed for the amide I and II bands was mostly due to the vibrational interactions among peptide groups in the chain and through hydrogen bonds [30]. Among them, the peak at 1694 cm^−1^ was the β-turn conformation in the amide I band [31], and the peaks at 1620 cm^−1^, 1516 cm^−1^, and 1227 cm^−1^ were the stretches of the vibration absorption peaks of amide I-III bands in the β-sheet structure, respectively, which was consistent with the literature [32]. There was no significant shift in the characteristic peaks of the silk fibers obtained from factory all-age artificial diet feeding, and no new peaks appeared, indicating that the main chemical structure of the two groups of silk was the same.

The amide I band of silk fibroin fibers was further deconvoluted and integrated with peaks, and the fitting results of the peaks are shown in Figure 2b–d. The content of the β-sheet structure of silk fibroin fibers after factory all-age artificial diets was slightly lower than that of the mulberry leaf group. This may be due to the fact that the intake of artificial feed hinders the transformation of α-helix and random coil into β-sheet, resulting in a decrease in β-sheet structures.

**Figure 3:**
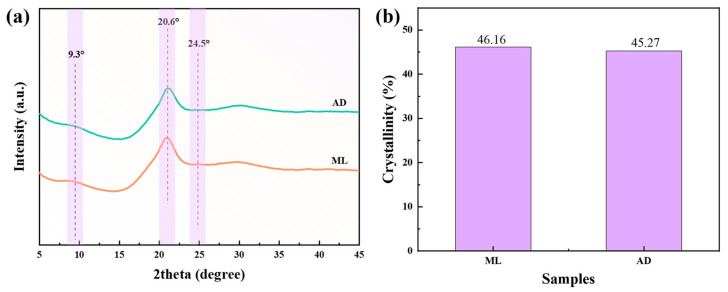
(**a**) XRD of silk fibers with two different feeding methods, (**b**) degree of crystallinity.

The changes in the crystalline structure of the silk fibers obtained by artificial feed being given to silkworms are shown in Figure 3. According to the positions of the two groups of silk diffraction peaks, the silk crystal diffraction peaks were detected at about 9.3, 20.6, and 24.5°. Moreover, the shapes and positions of the two sets of X-ray diffraction curves were almost identical. A distinct peak at 20.6° and two minor peaks at around 9.3 and 24.5° in the XRD spectrum were attributed to the predominant β-sheet crystalline structure of silk fibroin fibers [33,34]. MDI Jade was used to fit the peaks and calculate their crystallinity. The crystallinity of ML and AD was 46.16% and 45.27%, respectively. The crystal structure of the two groups was still dominated by a β-sheet structure, which was consistent with the above FTIR analysis conclusion.

### 2.3. Effect of Factory All-Age Artificial Diet Feeding on the Amino Acid Composition of Silk Fibers

Silk protein contains 18 kinds of amino acids. The type and content of amino acids often affect the structure of silk, but also, to a certain extent, they determine the basic properties of silk. In this experiment, acid hydrolysis was used to determine the content of various amino acids in the two groups of filaments, and tryptophan was destroyed in this process, so Table 1 presents the results of the determination of only 17 amino acids. As seen in the table, there was no significant difference in the amino acid composition of fibroin between feeding methods. Silk proteins had high glycine, alanine, and serine contents, accounting for about 75% of the total amino acid composition. In the crystalline region, the highly repetitive hexapeptide sequence GAGAGS (amino acids Gly-Ala-Gly-Ala-Gly-Ser) is considered to be the building block of the β-sheet microcrystalline region in silk proteins [35]. In the previous literature, it was reported that glycine and alanine are predominantly located in the crystalline region and that changes in the ratio of these two amino acids influence the crystal structure of silk proteins [36]. The calculated results showed that the ratio was slightly lower in the ML group, and fibroin proteins were more likely to form crystal structures. Another important factor affecting the crystallization process is the ratio between amino acid residues in the small and large side chains. The large side chain amino acid has a high molecular weight and is difficult to fold, which prevents the close arrangement of the peptide chains when the ratio is high, resulting in a coiled molecular conformation [37,38]. Tyrosine and phenylalanine are large sidearm amino acids, mainly located in the amorphous region, with a share of 11.37% and 11.22% in the ML and AD groups, respectively.

Furthermore, amino acids can be categorized into hydrophilic and hydrophobic amino acids. Hydrophilic amino acids are amino acids that are hydrophilic in water, such as aspartic acid, threonine, serine, glutamic acid, tyrosine, lysine, etc., also known as polar amino acids. The percentage of polar amino acids in the ML and AD groups was 30.82% and 30.30%, respectively. The common hydrophobic amino acids mainly included seven amino acids such as glycine, alanine, and isoleucine, and the proportion of them in the ML and AD groups was 67.45% and 67.96%, respectively.

### 2.4. Effect of Factory All-Age Artificial Diet Feeding on the Mechanical Properties of Silk Fibers

**Figure 4:**
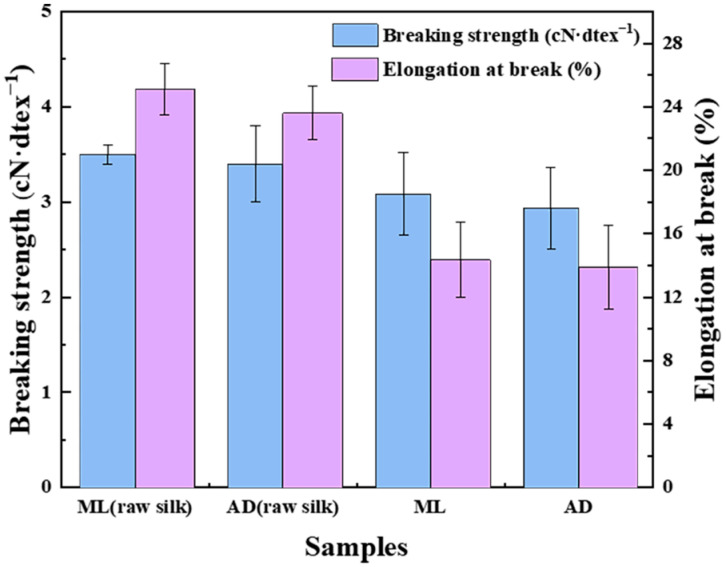
Mechanical properties of silk fibers with two different feeding methods.

It is well known that silk is a semi-crystalline biopolymer material, with crystalline regions formed by a β-sheet embedded in an amorphous protein matrix formed by a random coil. The strength of the silk fiber depends mainly on the crystalline zone, while the amorphous zone accounts for its extensibility [39]. The test results of the breaking strength and elongation at break of silk before and after degumming are shown in Figure 4. In degummed silk fibers without the support of the outer layer of silk glue, the cocoon filaments were scattered between the monofilament. At the same time, the covalent bonds between the fibers were weakened, so the breaking strength and elongation at break of the silk fibroin fibers decreased after degumming. Surprisingly, the strength of the silk fibers in the AD group was not much different from that of the ML group, due to their amino acid content and crystalline structure essentially being the same. The extensibility was 14.35% and 13.88% in the ML and AD groups, respectively. This was due to the higher proportion of large side chain amino acids in the ML group. However, there was no significant difference overall. It should be noted that there are inconsistencies in the results of silk fiber strength obtained by the factory all-age artificial diet feeding test on the market, which may be due to the results of different feed varieties.

### 2.5. Effect of Factory All-Age Artificial Diet Feeding on Hygroscopic Properties of Silk Fibers

**Figure 5:**
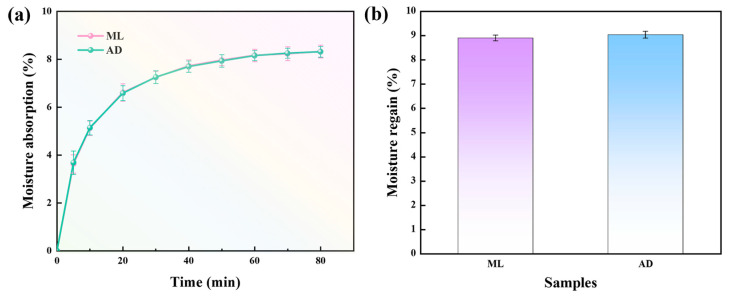
Hygroscopic properties of silk fibers with two different feeding methods. (**a**) Moisture absorption, (**b**) moisture regain.

In addition to the temperature and humidity of external conditions, the factors affecting the hygroscopicity and moisture regain of fiber are also related to its internal factors such as hydrophilic group, crystallinity, specific surface area, and so on. The test results of moisture absorption and moisture regain of silk fibroin fibers are shown in Figure 5. It can be seen from Figure 5a that the moisture absorption curves of the two groups of silk fibroin fibers were the same in each period. In the first 20 min, the moisture absorption first increased rapidly, and then, the upward trend slowed down and tended to be saturated after 60 min. The reason for the hygroscopic phenomenon is the presence of amorphous regions and hydrophilic amino acids in the internal structure of silk fibroin fibers. Hydrophilic functional groups such as amino groups and carboxyl groups in hydrophilic amino acids can form hydrogen bonds with water molecules, which enhances their ability to interact with water. However, according to “Section 2.3. Effect of Factory All-Age Artificial Diet Feeding on the Amino Acid Composition of Silk Fibers”, it can be found that the proportion of hydrophilic amino acids is smaller, the proportion of hydrophobic amino acids is greater, and the non-polar amino acids are the main components of silk fibroin fibers. Moreover, there is a crystalline region where the peptide chains are arranged neatly and tightly, and it is difficult for water molecules to enter, so the moisture absorption rate is less than 9%. Moisture regain also reflects the moisture absorption capacity of the fibers to a certain extent. As can be seen from Figure 5b, the AD group also had the same moisture regain rate as the ML group.

### 2.6. Effect of Factory All-Age Artificial Diet Feeding on Thermal Stability of Silk Fibers

**Figure 6:**
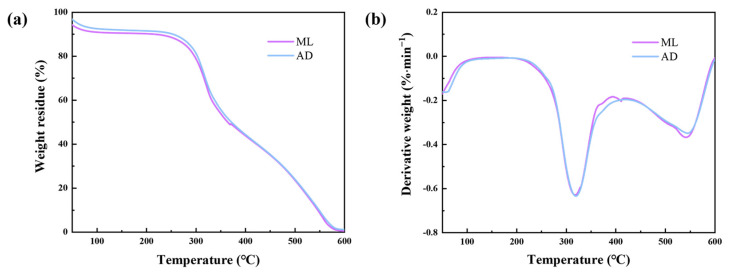
TG and DTG curves of silk fibers with two different feeding methods. (**a**) TG, (**b**) DTG.

The thermal properties of silk fibroin fibers in ML and AD were studied to further understand the effect of feed feeding. The thermogravimetric (TG) and differential thermogravimetric (DTG) curves of two sets of silk fibers from room temperature to 600 °C were obtained, and the results are shown in Figure 6. The thermal degradation characteristics of the two groups of silk fibroin samples were almost identical, which meant that different feeding methods did not cause significant changes in the thermal degradation process. An initial weight drop was observed at about 100 °C, and the main factor of mass loss was the evaporation of moisture inside the silk fibers. From 280 °C, the temperature continued to rise, which intensified the vibration of the internal groups of the silk fiber, the carboxyl and amino groups were gradually destroyed and decomposed into gas overflow, resulting in a sharp drop in the percentage of weight residue. The DTG curve reflects the change in the rate of mass loss with temperature. The figure showed that the two groups of samples had a large thermal degradation peak near 320 °C, the weight loss was mainly caused by the breakage of peptide bonds and side groups, and the silk fiber was violently decomposed [35]. A small degradation peak appeared at around 555 °C, and the degradation of silk fibers was slowed down. According to the test results, the thermal stability of silk fibers did not change significantly after artificial feed feeding.

### 2.7. Effect of Factory All-Age Artificial Diet Feeding on In Vitro Degradation of Silk Fibers

**Figure 7:**
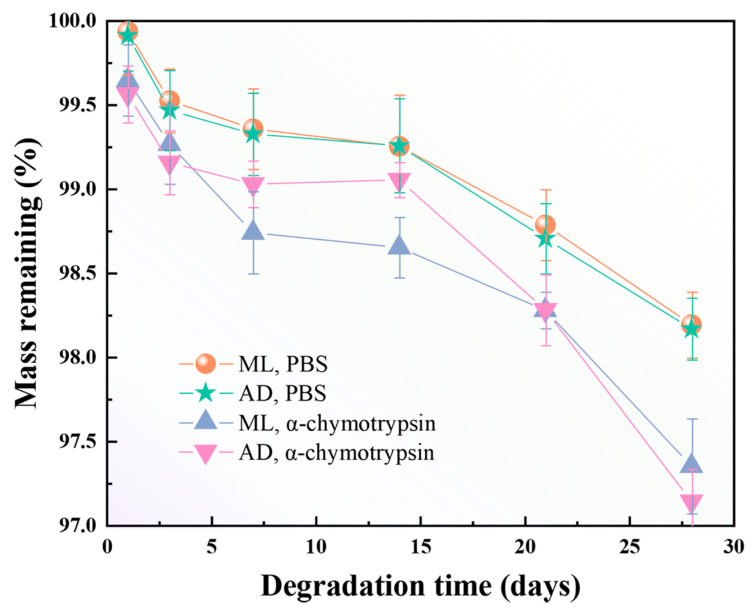
Enzymatic and PBS degradation curves of silk fibers with two different feeding methods.

In recent years, with the development of tissue engineering and the great potential of silk protein-based biomaterials in biomedical applications, it has become particularly important to explore the enzymatic degradation properties of silk protein materials. To facilitate later purification and application, the enzyme hydrolysis method with efficient and mild action was used in this case. Some in vitro studies on the degradation behavior of two groups of filamentous protein fibers were carried out using α-chymotrypsin. As can be seen in Figure 7, the residual mass of degummed silk fibers treated with PBS buffer and chymotrypsin solution decreased gradually with the increase in treatment time. A comparison of silk fibers prepared on the basis of different feeding methods showed that AD and ML had the same degree of reduction in silk residual mass. The rate of degradation was analyzed to be closely related to the structure and molecular weight [40,41] of filamentous fibrous proteins. This implies that aa non-dense structure and low molecular weight have a better ability to increase the rate of degradation of silk fibers. Soluble silk fiber has been reported to be degraded by α-chymotrypsin [42]. However, the ML and AD groups still had more than 97% mass residue after 28d of enzymatic hydrolysis. This shows that α-chymotrypsin has no serious effect on the degradation of silk fibers, which is in agreement with descriptions in the literature [43]. In fact, the rate of degradation is dependent on the concentration of the protease used, with larger concentrations leading to faster degradation. To increase the in vitro degradation of silk fibers, the concentration of the enzyme solution can be increased or other types of proteases can be selected.

**Figure 8:**
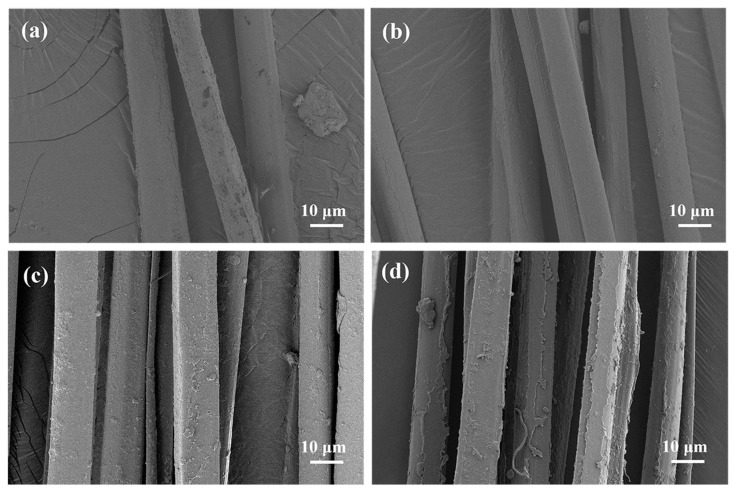
SEM image of silk fiber degradation process. (**a**) ML group after 14 d, (**b**) AD group after 14 d, (**c**) ML group after 28 d, (**d**) AD group after 28 d.

The surface of the silk fibers was damaged to varying degrees after exposure to α-chymotrypsin solution for 14 and 28 d. The results are shown in Figure 8. A closer look at Figure 8a,b shows that after 14 d of degradation, the surface of both groups of silk fibers showed fine traces of corrosion. The fibers of the ML group showed splitting, while the surfaces of the fibers of the AD group were relatively smooth. As the time of enzymatic degradation increased, the fiber surface roughening became more severe. As shown in Figure 8c,d, a large number of solubilized cracked particles appeared on the surface of the silk fibers. The AD group had its most severe erosion after 28 d. This was in general agreement with the results of the above analysis of the degradation rate of silk fibers after treatment with α-chymotrypsin solution.

## 3. Materials and Methods

### 3.1. Materials

The raw materials for this experiment were silkworm cocoons with two feeding methods, one of which was fed on mulberry leaves from Haian, Jiangsu Province, and the other was fed on a factory all-age artificial diet from the Zhejiang Babei group. The α-chymotrypsin for the in vitro degradation tests was purchased from Shanghai McLean Biochemical Science and Technology Co., Ltd., and the phosphate-buffered saline solution (PBS) was provided by Yida Technology Co., Ltd (Quanzhou, China). Deionized water was used throughout the process.

### 3.2. Preparation of Silk Fibroin Fibers

**Figure 9:**
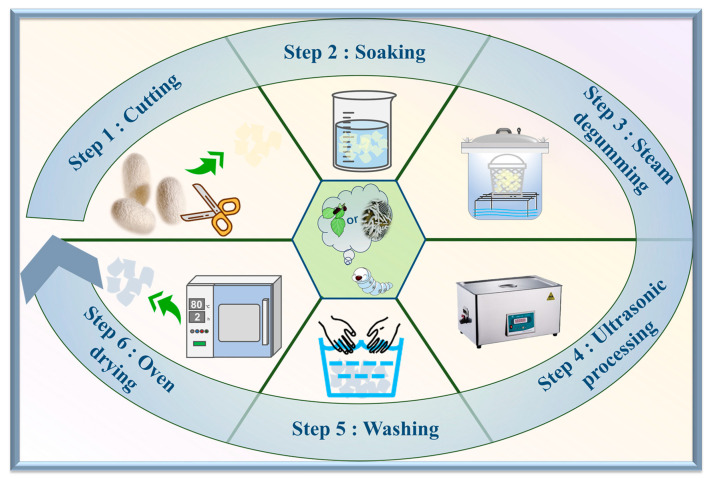
Steam degumming process.

The preparation process of silk fibroin fibers is shown in Figure 9. The steam degumming method was used for the acquisition of silk fiber, which was used as the material in this study. First of all, silkworm cocoons were torn apart into three layers to facilitate complete degumming. The deionized water-soaked cocoon shells were then subjected to degumming in a modified pressure cooker (XFS-280CB, Xinfeng Medical Apparatus, Zhejiang, China, a pressure meter was equipped on the cover lib) at 125 °C for 90 min [44]. Finally, the degummed cocoon shells were ultrasonically cleaned and then rinsed in warm water and dried to obtain the silk fibroin sample.

### 3.3. Measurement and Characterization

#### 3.3.1. Morphological Observation

The surface morphology of the silk fibroin specimens was scanned and observed using an Ultra55 scanning electron microscope (Carl Zeiss, Oberkochen, Germany) after gold palladium coating at an accelerating voltage of 3 kV with a magnification of 1000 times.

#### 3.3.2. Fourier Transform Infrared Spectroscopy Test

The Attenuating Total Reflection Fourier transform infrared (ATR-FTIR) spectra of degummed silk fibroin fibers were obtained using a Nicolet 5700 spectrometer (Thermo Nicolet, Shanghai, China) within a wavelength range of 4000–400 cm^−1^ with a spectral resolution of 4 cm^−1^.

#### 3.3.3. X-ray Diffraction Test

The crystalline structure was tested using an X’TRA X-ray diffractometer with an X-ray tube that produces monochromatic Cu-Kα radiation. When scanning, the SF fibers were cut into powders, placed on the sample stage, and scanned from 5° to 50° at a scanning speed of 2(°)/min under the conditions of 40 kV and 40 mA.

#### 3.3.4. Amino Acid Composition Test

The amino acid composition of the hydrolysates was measured with a Hitachi L8900 Amino Analyzer (Hitachi Company, Tokyo, Japan). The two kinds of silk fibroin fiber samples were hydrolyzed in 10 mL of 6 mol/L HCL in a vacuum atmosphere at 110 °C for 24 h and then filtered. Subsequently, each sample was deacidified and dried on a rotary evaporator at 45 °C and filtered again by adding buffer solution. Three samples of SF fibers from each group were tested to obtain the average values.

#### 3.3.5. Mechanical Property Test

The breaking strength and elongation at break of the degummed silk fibers were determined by the YG004 electronic strength machine (Zhongxian Testing Equipment, Changzhou, China) with a gauge length of 25 mm at a stretching speed of 100 mm/min. All specimens were subjected to experiments at (20 ± 2) °C and (65 ± 4) % relative humidity (RH). Thirty samples of degummed silk fibers were tested in each group, and the results were averaged.

#### 3.3.6. Hygroscopic Test

The moisture absorption rate of silk fibers indicates the percentage of water absorbed compared to its moisture-free weight. The dried samples were weighed and quickly transferred to a desiccator containing a saturated copper sulphate solution, where they were subjected to hygroscopic experiments at a temperature of (20 ± 2) °C and a relative humidity range of (65 ± 4)%. In the moisture regain test, the sample was subjected to standard conditions for 12 h. The mass of each set of specimens was recorded and calculated according to the following formulas:

(1) $$\mathrm{Moisture}\;\mathrm{absorption}\;\mathrm{rate}\;(\%)=\frac{m_{2}-m_{0}}{m_{0}}\times 100,$$

(2) $$\mathrm{Moisture}\;\mathrm{regain}\;\mathrm{rate}\;(\%)=\frac{m_{1}-m_{0}}{m_{0}}\times 100,$$

where *m*_2_ is the mass after moisture absorption, *m*_1_ is the initial mass under standard conditions, and *m*_0_ is the mass of drying to constant weight.

#### 3.3.7. Thermal Stability Test

Thermogravimetric studies of the silk fibroin powders were conducted by a thermogravimetric analyzer (TGA550, TA, New Castle, DE, USA). The thermograms were obtained under a nitrogen atmosphere (60 mL/min) at a uniform heating rate of 10 °C/min in the temperature range of 50–650 °C.

#### 3.3.8. In vitro Enzymatic Degradability Test

Centrifuge tubes were first sterilized in an autoclave for 20 min. Each sample was then individually placed in a 50 mL centrifuge tube and sterilized by UV irradiation for 30 min. After complete sterilization, 1 mg/mL of α-chymotrypsin and 30 mL of PBS buffer were added to the centrifuge tubes in an ultra-clean bench. Finally, they were placed in a constant-temperature shaker at 37 °C for degradation, and the buffer was replaced with fresh buffer every 7 d. Each group of samples was dried and weighed at 1, 3, 7, 14, 21, and 28 d. The mass residual rate was calculated according to Equation (3).

(3) $$\mathrm{Mass}\;\mathrm{residual}\;\mathrm{rate}\;(\%)=100-\frac{W_{1}\;-W_{2}}{W_{1}}\times 100$$

where *W*_1_ is the dry weight before degradation and *W*_2_ is the dry weight after degradation.

#### 3.3.9. Statistical Analysis

SPSS 27 was used for significant difference analysis, and the independent-sample T test was used for comparison between the AD group and the ML group. The *p*-values were more than 0.05, and there was no significant difference. The data in this study were presented as the mean and standard deviation (SD).

## 4. Conclusions

The raw silk obtained using the two feeding methods was degummed to obtain silk fibroin fibers. The secondary structure, crystalline structure, and thermal properties of silk fibroin fibers were characterized and analyzed with the help of FTIR, XRD, amino acid composition analysis, and thermogravimetric analysis. Comparing the differences between the physical properties of silk obtained by feeding silkworms with factory all-age artificial diets and mulberry leaves led to the following conclusions: The FTIR and XRD curves of the AD group were not significantly different from those of the ML group, resulting in their mechanical and hygroscopic properties being the same. And the difference in amino acid composition and content between the two groups was very small. The surface of the silk fibers was rougher in the AD group after 28 days of incubation in α-chymotrypsin solution, and there was not much difference in degradation rates between the two groups. The above results indicate that a factory all-age artificial diet for silkworms is feasible. If we want to further promote the transformation and development of the sericulture industry, researchers also need to prepare factory all-age artificial diet-regenerated fibroin protein materials, such as films, hydrogels, scaffolds, etc., to expand the artificial feeder cocoons of silkworms in the field of biomedicine and other new fields.

## Figures and Tables

**Table 1:** Differences in amino acid composition of silk fibers with two different feeding methods.

Types of Amino Acids	Relative Content of 17 AA (%)		Change (ML-AD)
	ML Group	AD Group	
Aspartic acid	2.46	2.47	−0.01
Threonine	1.21	1.22	−0.01
Serine	12.86	12.47	0.39
Glutamic acid	2.15	2.21	−0.06
Proline	0.77	0.74	0.03
Glycine	35.65	36.20	−0.55
Alanine	25.81	25.72	0.09
Cystine	0.96	1.00	−0.04
Valine	2.95	2.95	0.00
Methionine	0.15	0.15	0.00
Isoleucine	0.88	0.91	−0.03
Leucine	0.79	0.79	0.00
Tyrosine	10.16	9.98	0.18
Phenylalanine	1.21	1.23	−0.02
Lysine	0.79	0.75	0.04
Histidine	0.24	0.25	−0.01
Arginine	0.94	0.94	0.00

## Data Availability

The data presented in this study are available on request from the corresponding author.

## References

[1] Yi Q., Zhao P., Wang X., Zou Y., Zhong X., Wang C., Xiang Z., Xia Q. (2013). Shotgun proteomic analysis of the *Bombyx mori* anterior silk gland: An insight into the biosynthetic fiber spinning process. Proteomics.

[2] Nguyen T.P., Nguyen Q.V., Nguyen V., Le T., Huynh V.Q.N., Vo D.N., Trinh Q.T., Kim S.Y., Le Q.V. (2019). Silk Fibroin-Based Biomaterials for Biomedical Applications: A Review. Polymers.

[3] Shao Z., Frid V. (2002). Surprising strength of silkworm silk. Nature.

[4] Teramoto H., Kojima K., Iga M., Yoshioka T. (2023). Unique Material Properties of *Bombyx mori* Silk Fiber Incorporated with 3-Azidotyrosine. Biomacromolecules.

[5] Jin K.Y., Wan K.S., Young K.K., Seok K.C., Chul U.I. (2023). Structural Characteristics and Properties of Cocoon and Regenerated Silk Fibroin from Different Silkworm Strains. Int. J. Mol. Sci..

[6] Lu Q., Zhang B., Li M., Zuo B., Kaplan D.L., Huang Y., Zhu H. (2011). Degradation mechanism and control of silk fibroin. Biomacromolecules.

[7] Debari M.K., King C.I., Altgold T.A., Abbott R.D. (2021). Silk Fibroin as a Green Material. ACS Biomater. Sci. Eng..

[8] Lamboni L., Gauthier M., Yang G., Wang Q. (2015). Silk sericin: A versatile material for tissue engineering and drug delivery. Bio-Technol. Adv..

[9] Agarwal Y., Rajinikanth P., Ranjan S., Tiwari U., Balasubramnaiam J., Pandey P., Arya D.K., Anand S., Deepak P. (2021). Curcumin loaded polycaprolactone-/polyvinyl alcohol-silk fibroin based electrospun nanofibrous mat for rapid healing of diabetic wound: An in-vitro and in-vivo studies. Int. J. Biol. Macromol..

[10] Sina A.S., Rouhollah M.A., Parisa A., Asieh H.T. (2022). Amniotic membrane/silk fibroin-alginate nanofibrous scaffolds containing Cu-based metal organic framework for wound dressing. Int. J. Polym. Mater. Polym. Biomat..

[11] Varshney N., Sahi A.K., Poddar S., Mahto S.K. (2020). Soy protein isolate supplemented silk fibroin nanofibers for skin tissue regeneration: Fabrication and characterization. Int. J. Biol. Macromol..

[12] Ding Z., Cheng W., Mia M.S., Lu Q. (2021). Silk Biomaterials for Bone Tissue Engineering. Macromol. Biosci..

[13] Mina A., Atefeh S., Somaye A., Mohammad M. (2022). A hydrogel-fiber-hydrogel composite scaffold based on silk fibroin with the dual-delivery of oxygen and quercetin. Biotechnol. Bioeng..

[14] Lu L., Liu X., Sun Y., Wang S., Liu J., Ge S., Wei T., Zhang H., Su J., Zhang Y. (2024). Silk-Fabric Reinforced Silk for Artificial Bones. Adv. Mater..

[15] Hamamura Y. (1959). Food Selection by Silkworm Larvæ. Nature.

[16] Qin D., Wang G., Dong Z., Xia Q., Zhao P. (2020). Comparative Fecal Metabolomes of Silkworms Being Fed Mulberry Leaf and Artificial Diet. Insects.

[17] Li J., Deng J., Deng X., Liu L., Zha X. (2023). Metabonomic Analysis of Silkworm Midgut Reveals Differences between the Physiological Effects of an Artificial and Mulberry Leaf Diet. Insects.

[18] Zhou Z., Yang H., Chen M., Lou C., Zhang Y., Chen K., Wang Y., Yu M., Fang Y., Li J. (2008). Comparative proteomic analysis between the domesticated silkworm (*Bombyx mori*) reared on fresh mulberry leaves and on artificial diet. J. Proteome Res..

[19] Chen X., Ye A., Wu X., Qu Z., Xu S., Sima Y., Wang Y., He R., Jin F., Zhan P. (2022). Combined analysis of silk synthesis and hemolymph amino acid metabolism reveal key roles for glycine in increasing silkworm silk yields. Int. J. Biol. Macromol..

[20] Dong H., Zhang S., Tao H., Chen Z., Li X., Qiu J., Cui W., Sima Y., Cui W., Xu S. (2017). Metabolomics differences between silkworms (*Bombyx mori*) reared on fresh mulberry (Morus) leaves or artificial diets. Sci Rep.

[21] Wang Y., Shu Q., Gu H., Feng P., Dai M., Zhu Q., Liu W., Dai Y., Li F., Li B. (2023). Effects of different diets on the growth and development of young silkworms. J. Asia-Pac. Entomol..

[22] Shu Q., Wang Y., Gu H., Zhu Q., Liu W., Dai Y., Li F., Li B. (2023). Effects of artificial diet breeding on intestinal microbial populations at the young stage of silkworm (*Bombyx mori*). Arch. Insect Biochem. Physiol..

[23] Li J., Chen C., Zha X. (2022). Midgut and Head Transcriptomic Analysis of Silkworms Reveals the Physiological Effects of Artificial Diets. Insects.

[24] Wu X., Chen X., Ye A., Cao J., He R., Pan M., Jin F., Ma H., Zhou W. (2022). Multi-tissue metabolomic profiling reveals po-tential mechanisms of cocoon yield in silkworms (*Bombyx mori*) fed formula feed versus mulberry leaves. Front. Mol. Biosci..

[25] Jiang L., Huang T., Liu Q., Zhong S., Shen D., Chen A., Zhao Q. (2023). Transcriptome analysis of anorexic and preferred silkworms (*Bombyx mori*) on artificial diet. Comp. Biochem. Physiol. Part D Genom. Proteom..

[26] Tao S., Wang J., Liu M., Sun F., Li B., Ye C. (2021). Haemolymph metabolomic differences in silkworms (*Bombyx mori* L.) under mulberry leaf and two artificial diet rearing methods. Arch. Insect Biochem. Physiol..

[27] Qu J., Feng P., Zhu Q., Ren Y., Li B. (2021). Study on the Effect of Stretching on the Strength of Natural Silk Based on Different Feeding Methods. ACS Biomater. Sci. Eng..

[28] Xu X., Yao X., Jiang K., Zhou Y., Lu W., Jiang W., Wang X. (2022). Novel ultrasonic-assisted cleaner technology for cocoon brushing at low temperature. J. Clean. Prod..

[29] Lee K.I., Wang X., Guo X., Yung K., Fei B. (2017). Highly water-absorbing silk yarn with interpenetrating network via in situ polymerization. Int. J. Biol. Macromol..

[30] Miyazawa T., Blout E.R.J. (1961). The Infrared Spectra of Polypeptides in Various Conformations: Amide I and II Bands. J. Am. Chem. Soc..

[31] Paquet-Mercier F., Lefevre T., Auger M., Pezolet M. (2013). Evidence by infrared spectroscopy of the presence of two types of β-sheets in major ampullate spider silk and silkworm silk. Soft Matter.

[32] Ling S., Qi Z., David P.K., Shao Z., Chen X. (2011). Synchrotron FTIR microspectroscopy of single natural silk fibers. Biomacromolecules.

[33] Yeon-su B., In-chul U. (2021). Effects of Fabrication Conditions on Structure and Properties of Mechanically Prepared Natural Silk Web and Non-Woven Fabrics. Polymers.

[34] Yeong C.Y., Jin J.M., Byung-Dae P., Chul U.I. (2023). Fabrication, Structure, and Properties of Nonwoven Silk Fabrics Prepared with Different Cocoon Layers. Int. J. Mol. Sci..

[35] Zhao M., Qi Z., Tao X., Chad N., Hu X., Lu S. (2021). Chemical, Thermal, Time, and Enzymatic Stability of Silk Materials with Silk I Structure. Int. J. Mol. Sci..

[36] Sen K., Babu K.M. (2004). Studies on Indian silk. I. Macrocharacterization and analysis of amino acid composition. J. Appl. Polym. Sci..

[37] Cheng L., Huang H., Chen S., Wang W., Dai F., Zhao H. (2017). Characterization of silkworm larvae growth and properties of silk fibres after direct feeding of copper or silver nanoparticles. Mater. Des..

[38] Reddy N., Yang Y. (2012). Investigation of the Structure and Properties of Silk Fibers Produced by *Actias lunas*. J. Polym. Environ..

[39] Fang G., Tang Y., Qi Z., Yao J., Shao Z., Chen X. (2017). Precise correlation of macroscopic mechanical properties and microscopic structures of animal silks-using Antheraea pernyi silkworm silk as an example. J. Polym. Environ..

[40] Koperska M., Pawcenis D., Bagniuk J., Zaid M., Missori M., Łojewski T., Łojewska J. (2014). Degradation markers of fibroin in silk through infrared spectroscopy. Polym. Degrad. Stabil..

[41] Rajkhowa R., Hu X., Tsuzuki T., Kaplan D.L., Wang X. (2012). Structure and Biodegradation Mechanism of Milled *Bombyx mori* Silk Particles. Biomacromolecules.

[42] Li M., Ogiso M., Minoura N. (2003). Enzymatic degradation behavior of porous silk fibroin sheets. Biomaterials.

[43] Thidarat W., Johnston B.F., Philipp S.F. (2018). Degradation Behavior of Silk Nanoparticles—Enzyme Responsiveness. ACS Biomater. Sci. Eng..

[44] Wang R., Zhu Y., Shi Z., Jiang W., Liu X., Ni Q. (2018). Degumming of raw silk via steam treatment. J. Clean. Prod..

